# A drink before suicide: analysis of the Queensland Suicide Register in Australia

**DOI:** 10.1017/S2045796020000062

**Published:** 2020-01-24

**Authors:** Kairi Kõlves, Yu Wen Koo, Diego de Leo

**Affiliations:** Australian Institute for Suicide Research and Prevention, World Health Organization Collaborating Centre for Research and Training in Suicide Prevention, School of Applied Psychology, Griffith University, Mount Gravatt, Queensland, Australia

**Keywords:** Blood alcohol concentration, medicine, substances, suicide

## Abstract

**Aims:**

Previous studies analysing blood alcohol concentration (BAC) at the time of suicide have primarily focused on sociodemographic factors. Limited research has focused on psychosocial factors and co-ingestion of other substances to understand the mechanisms of how alcohol contributes to death by suicide. The aim was to examine time trends, psychosocial factors related to acute alcohol use and co-ingestion of alcohol and other substances before suicide.

**Methods:**

The Queensland Suicide Register in 2004–2015 was utilised and analysed in 2019. The cut-off point for positive BAC was set at ⩾0.05 g/dl. Substances were categorised as medicines, illegal drugs and other. Medicines were coded by the Anatomical Therapeutic Chemical (ATC) classification system. Joinpoint regression, univariate odds ratios, age and sex-adjusted odds ratios and Forward Stepwise logistic regression were performed.

**Results:**

BAC information was available for 6744 suicides, 92% of all cases in 2004–2015. The final model showed that independent factors distinguishing BAC+ from BAC− were: age group 25–44 years, Australian Indigenous background, being separated or divorced, hanging, diagnosis of substance use, lifetime suicidal ideation, relationship and interpersonal conflict, not having psychotic and other psychiatric disorder, and no nervous system drugs or any other substances in blood at the time of suicide.

**Conclusions:**

Our findings suggest that people who die by suicide while under the influence of alcohol are more likely to be under acute stress (e.g. separation) and not have earlier psychiatric conditions, except substance use. This highlights the importance of more strict alcohol policies, but also the need to improve substance use treatment.

## Introduction

In the 2016 Global Burden of Disease Study, alcohol use was the leading risk factor of death in the age group 15–49 years with suicide being the third leading alcohol attributable cause of death (Griswold *et al*., 2018). By the World Health Organization's estimates, approximately 800 000 people died by suicide in 2016, which equates to an age-standardised suicide rate of 10.5 per 100 000 population (World Health Organization, n.s.). In Australia, approximately 3000 people die by suicide annually since 2014 with age-standardised suicide rates showing an increase since 2006, from 10.2 to 12.2 per 100 000 population in 2018 (Australian Bureau of Statistics, 2019*a*). By the Australian Burden of Disease Study 2015, alcohol use contributed to 14% of the burden due to suicide (Australian Institute of Health and Welfare, 2019). While both abuse and acute alcohol use have been found to be predictors of suicidal behaviour, alcohol use is rarely addressed in Australian suicide research and prevention activities (Witt and Lubman, 2018). The current study will focus on acute alcohol use prior to suicide in Australia.

### Acute alcohol use and suicide

Acute alcohol use has been shown to be an independent predictor of suicidal behaviour (Cherpitel *et al*., 2004; Pompili *et al*., 2010; Conner *et al*., 2014). A meta-analysis of 92 studies (1979–2012) found that acute alcohol use contributed to a third of suicides (Anestis *et al*., 2014; Conner, 2015), ranging between 9.5% in Thailand (Narongchai and Narongchai, 2006) and 74.2% in Slovenia (Bilban and Skibin, 2005). Several different mechanisms that may increase the risk of suicidal behaviours while under the influence of alcohol have been proposed. The increase in impulsivity and aggression as a result of alcohol use is one of the most frequently suggested link in the literature (Brady, 2006; Pompili *et al*., 2010; Turecki, 2014). However, alcohol intoxication can also promote depressive thoughts and feelings of hopelessness, especially if people are predisposed and have depression, and alcohol might be used for self-medication (Cherpitel *et al*., 2004; Holmgren and Jones, 2010). In addition, cognitive constriction through alcohol myopia may contribute to suicidal behaviour as people under the influence of alcohol tend to overemphasise recent adverse life events; therefore, alcohol intoxication might be critical if the person has recently lost a job or experienced a separation from a relationship (Brady, 2006). Furthermore, alcohol disinhibits barriers, including potential fear of death, which may also increase the risk of suicidal behaviour (Pompili *et al*., 2010; Anestis *et al*., 2014). Additionally, alcohol may interact with other drugs and increase their lethality (Cherpitel *et al*., 2004; Holmgren and Jones, 2010; Anestis *et al*., 2014).

There are some large-scale studies from the USA, analysing blood alcohol concentration (BAC) at the time of suicide; however, they have primarily focused on sociodemographic factors, and there is rather limited research focussing on psychosocial factors including life events and psychiatric conditions. Only a very small number of studies have analysed co-ingestion of alcohol with other substances to understand the mechanisms of how alcohol contributes to death by suicide. Therefore, we aim to:

1. analyse the rates and changes in the prevalence of acute alcohol use (BAC+) in people who died by suicide in Queensland, Australia;
2. identify psychosocial factors related to acute alcohol use in suicides; and
3. examine the co-ingestion of alcohol and other substances before suicide.

## Methods

### Context

Queensland is located in North East of Australia, being the second largest (1 852 642 km^2^) and third most populous state; with a population of over 5 million people (Australian Bureau of Statistics, 2019*b*). By the 2016 Census of Population and Housing, 4% of the population of Queensland were Aboriginal and Torres Strait Islanders (2.8% of the all-Australian population) and 28.9% were born outside of Australia (33.3% for all Australia; Australian Bureau of Statistics, n.s.). Average age-standardised suicide rate was 14.4 per 100 000 in 2012–2016 (11.9 for all Australia; Australian Bureau of Statistics, 2019*a*).

### Data sources

The analyses used data from the Queensland Suicide Register (QSR), an active suicide surveillance system running since 1990, managed by the Australian Institute for Suicide Research and Prevention (AISRAP). Primary information sources of the QSR include

- police report of death to a Coroner (referred to coronial records as Form 1) including a narrative of circumstances of death (who the deceased person was, and how, when and where the person died),
- post-mortem autopsy report,
- toxicology report and
- Coroner's findings (Potts *et al*., 2016).

Documents were retrieved from the Queensland Coroners Court and from the National Coronial Information System (NCIS). The QSR procedures are approved by the Griffith University Human Research Ethics Committee (CSR/02/10/HREC) and the Justice Department Human Research Ethics Committee (CF/15/13188).

The availability of primary information sources has slightly changed between 1990 and 2015. Post-mortem autopsy reports represent an essential document for the inclusion of cases in the QSR, hence their availability has been close to 100% since the establishment of the QSR in 1990 (Potts *et al*., 2016). However, the police report (Form 1) and the toxicology report reached over 90% in 2004, therefore our analysis is using data from 2004 to 2015. Considering information about socio-demographic background, details about death (suicide method, concentration alcohol and drugs in blood, etc.), life events, psychiatric conditions comes from the primary information sources, their completeness is crucial for comparability over time.

The QSR scrutinises all cases of possible suicide to determine the level of probability that the death was due to suicide. Following a decision tree, cases are classified into three different levels: (1) beyond reasonable doubt, (2) probable and (3) possible (Potts *et al*., 2016). It is important to note that alcohol consumption prior to death is not a criterion in the classification process. For the present analyses, only cases falling into the categories of Beyond Reasonable Doubt and Probable were included.

The toxicology reports provide results of an analysis of any substances present in the circulatory, urinary and digestive systems of the deceased, such as alcohol, illicit drugs, medications and poisons. BAC is presented in the toxicology report and measured in g/100 ml ( = %). The cut-off point for BAC+ was set at 0.05 g/dl as care is needed when interpreting low levels due to the risk of bacterial production of ethanol in the body; especially when extensive trauma or decomposition has occurred (Kugelberg and Jones, 2007). In addition, BAC of 0.05 g/dl is the legal threshold level to define a state of intoxication in Australia. All substances in the system of the deceased individuals were initially categorised into three wider groups: medicines, illegal drugs and other substances (mainly pesticides and herbicides). Medicines were further coded by the Anatomical Therapeutic Chemical (ATC) classification system, considered as the gold standard for international drug utilisation monitoring and research (Norwegian Institute for Public Health, n.s.). The ATC classifies all medicines by the main therapeutic use of the main active ingredient, and the active substances are classified in a hierarchy with five different levels. If the medicine had multiple codes and the first main level located one of the codes into the nervous system (N), then the assumption was made that the medicine was used for the treatment of nervous system; if none of the codes was in N group, then the first code was used. After classification, in the current study, the medicines were further grouped into:

- Opioids (N2A)
- Other analgesics (N2B, N2C)
- Antipsychotics (N5A)
- Anxiolytics (N5B)
- Hypnotics/sedatives (N5C)
- Antidepressants (N6A)
- Other nervous system drugs (N1, N3, N4, N6B, N6D, N7)
- Other medicines (all other main groups).

### Statistical analysis

The *χ*^2^_trend_ was calculated to measure a change in the prevalence of BAC+ over the study period. Crude suicide rates were calculated for BAC+ and BAC− cases to observe changes in absolute terms (per 100 000 population). Population data were used in order to calculate the rates (Australian Bureau of Statistics, 2017). Joinpoint regression analyses were performed to test the changes in trends for BAC+ and BAC− by sex between 2004 and 2015. Joinpoint regression enables the identification of the best-fitting points where a statistically significant change in trend occurred and calculates the annual percentage change (APC) with the 95% confidence intervals (95% CI). Univariate odds ratios (OR) and age and sex-adjusted odds ratios (AdjOR) with 95% CI were calculated when comparing characteristics between BAC+ and BAC− cases. We performed a Forward Stepwise logistic regression model to identify the best-fitting model to predict BAC+ (compared to BAC−). A *χ*^2^ test shows the ability of these variables to distinguish between groups (BAC+ *v*. BAC−) relative to a constant only model. Nagelkerke's *R*^2^ presents the proportion of explained variance of the BAC by the independent variables in the model. The analyses were performed with the IBM SPSS version 25.0 and the Joinpoint Regression Program version 4.2.0.1 in 2019.

## Results

Between 2004 and 2015, 4.1% of the QSR cases were missing a toxicology report (not conducted/not available in the system). Due to several reasons (e.g., stage of body decomposition), it is not always feasible to measure BAC, so an additional 3.9% were missing BAC-level information. Therefore, our analysis included 92.0% of all cases (beyond reasonable doubt and probable) recorded in the QSR between 2004 and 2015, corresponding to 6744 suicide cases.

### Prevalence and rates of BAC+ and BAC− suicides

**Fig. 1.**
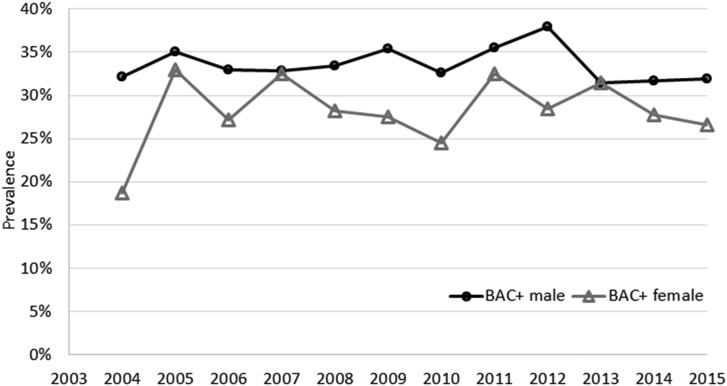
Prevalence of BAC+ in suicide cases at the time of death by sex in Queensland, 2004–2015.

In total, 32.3% of people who died by suicide had positive BAC at the time of death; this was significantly more likely to occur in males compared to females: 33.6 and 28.3%, respectively (OR = 1.28, 95% CI 1.13–1.45). During the study period, there were no significant linear changes in the prevalence for males (*χ*^2^_trend_ = 0.12, df = 1, *p* = 0.725) or females (*χ*^2^_trend_ = 2.42, df = 1, *p* = 0.623) (Fig. 1).

**Fig. 2.**
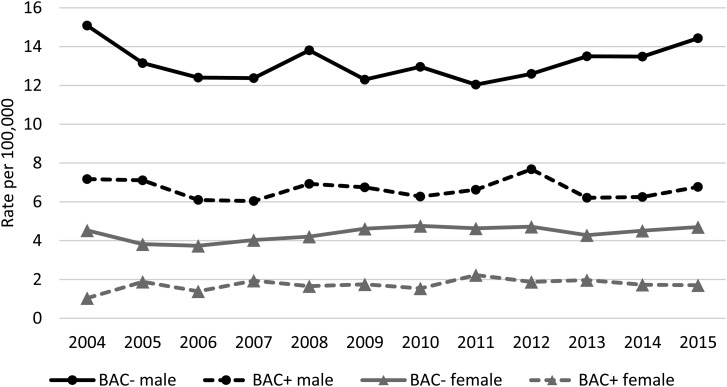
Crude rates of BAC+ and BAC− suicides at the time of death by sex in Queensland, 2004–2015.

Trends of crude suicide rates per 100 000 population for BAC+ and BAC− by sex showed relatively stable trends and Joinpoint regression analyses did not identify any significant changes or trends for BAC+ males or females (Fig. 2). Only for BAC− females, 2006 was identified as a joinpoint, which was followed by a significant upward trend in until 2015 (APC = 1.88%, *p* = 0.03).

### Sociodemographic characteristics

After adjusting for sex, young adults (25–44 years – used as the reference group) were most likely to be BAC+ at the time of death and older adults (65+) least likely (AdjOR = 0.24, 95% CI 0.20–0.29) (Table 1). Among Aboriginal and Torres Strait Islander Peoples who died by suicide, 56.3% were BAC+, with sex- and age-adjusted odds of BAC+ being 2.72-times higher (95% CI 2.21–3.36) compared to other Australians. Separated (AdjOR = 1.37, 95% CI 1.17–1.61) and divorced (AdjOR = 1.28, 95% CI 1.03–1.60) were more likely to be BAC+ compared to those who were married, and never married were less likely to be BAC+ (AdjOR = 0.81, 95% CI 0.70–0.94).

**Table 1.** BAC+ and BAC− by different demographic, psychosocial and suicide-related characteristics

	BAC+		BAC−			
	*N*	%	*N*	%	Crude OR (95% CI)	Adjusted^a^ OR (95% CI)
Sex						
Male	1727	79.2	3418	74.9	**1.28** (1.13–1.45)***	**1.29** (1.14–1.46)***
Female	453	20.8	1146	25.1	1 (RC)	1 (RC)
Age groups						
Below 25 years	309	14.2	674	14.8	0.72 (0.62–0.84)***	0.73 (0.62–0.85)***
25–44 years	1084	49.7	1702	37.3	1 (RC)	1 (RC)
45–64 years	664	30.5	1384	30.3	0.75 (0.67–0.85)***	0.76 (0.68–0.86)***
65 years and older	123	5.6	804	17.6	0.24 (0.20–0.30)***	0.24 (0.20–0.29)***
Suicide methods						
Poisoning by drugs/medicine	269	12.3	865	19.0	0.52 (0.48–0.57)***	0.63 (0.53–0.74)***
Poisoning by other means	240	11.0	420	9.2	0.97 (0.89–1.06)	1.07 (0.90–1.28)
Hanging or suffocation	1309	60.0	2215	48.5	1 (RC)	1 (RC)
Firearms or explosives	134	6.1	373	8.2	0.61 (0.54–0.68)*	0.78 (0.63–0.97)*
Other methods	228	10.5	691	15.1	0.56 (0.51–0.61)***	0.61 (0.52–0.72)***
Ethnicity						
Aboriginal and Torres Strait Islander	229	10.5	185	4.1	2.83 (2.32–3.47)***	2.72 (2.21–3.36)***
Unknown ethnicity	305	14.0	610	13.4	1.15 (0.99–1.33)	1.14 (0.98–1.32)
Other Australian	1646	75.5	3769	82.6	1 (RC)	1 (RC)
Marital status						
Single/never married	533	35.1	1654	36.2	0.99 (0.87–1.14)	0.81 (0.70–0.94)**
Married/*de facto*	765	24.4	1161	25.4	1 (RC)	1 (RC)
Separated	376	17.2	545	11.9	1.49 (1.28–1.75)***	1.37 (1.17–1.61)***
Divorced	152	7.0	287	6.3	1.15 (0.92–1.42)	1.28 (1.03–1.60)*
Widowed	32	1.5	194	4.3	0.36 (0.24–0.52)***	0.76 (0.51–1.14)
Unknown	322	14.8	723	15.8	0.96 (0.82–1.13)	0.90 (0.76–1.05)
Psychiatric disorders						
A diagnosed psychiatric disorder	979	44.9	2437	46.6	0.71 (0.64–0.79)***	0.74 (0.66–0.82)***
Unipolar depression	753	34.6	1768	38.7	0.84 (0.75–0.93)**	0.89 (0.80–1.00)*
Bipolar disorder	84	3.9	239	5.2	0.73 (0.56–0.94)*	0.75 (0.58–0.96)*
Anxiety disorders	143	6.6	390	8.5	0.75 (0.62–0.92)**	0.78(0.64–0.96)*
Psychotic disorders	63	2.9	358	7.8	0.35 (0.27–0.46)***	0.33 (0.25–0.43)***
Substance use disorders	207	9.5	255	5.6	1.18 (1.47–2.15)***	1.75 (1.45–2.12)***
Any other psychiatric disorder	122	5.6	349	92.4	0.72 (0.58–0.89)**	0.69 (0.56–0.86)**
An untreated mental health problems	791	36.3	1376	30.1	1.32 (1.19–1.47)***	1.25 (1.12–1.39)***
Lifetime suicide attempt	691	31.7	1409	30.9	1.04 (0.93–1.16)	1.02 (0.91–1.14)
Lifetime suicidal ideation	1052	48.3	1979	43.4	1.22 (1.10–1.35)***	1.20 (1.08–1.33)***
A physical condition	828	38.0	2312	49.3	0.60 (0.54–0.66)***	0.70 (0.63–0.78)***
Life events						
Relationship conflict	465	21.3	536	11.7	2.04 (1.78–2.34)***	1.90 (1.66–2.18)***
Relationship separation	643	29.5	1054	23.1	1.39 (1.24–1.56)***	1.29 (1.15–1.45)***
Bereavement	271	12.4	539	11.8	1.06 (0.91–1.24)	1.12 (0.95–1.31)
Familial conflict	219	10.0	397	8.7	1.17 (0.99–1.39)	1.12 (0.94–1.34)
Interpersonal conflict	160	7.3	182	4.0	1.91 (1.53–2.37)***	1.77 (1.42–2.20)***
Pending legal matters	224	10.3	376	8.2	1.28 (1.07–1.52)**	1.17 (0.98–1.39)
Financial problems	337	15.5	640	14.0	1.12 (0.97–1.29)	1.11 (0.96–1.29)
Recent/pending unemployment	223	10.2	372	8.2	1.29 (1.08–1.53)**	1.23 (1.03–1.47)*
Work/school problems (not financial)	142	6.5	338	7.4	0.87 (0.71–1.07)	0.81 (0.66–1.00)*
Child custody dispute	127	5.8	172	3.8	1.58 (1.25–2.00)***	1.47 (1.16–1.86)**
Childhood trauma	57	2.6	145	3.2	0.82 (0.60–1.12)	0.77 (0.56–1.05)
Sexual abuse	59	2.7	90	2.0	1.38 (0.99–1.93)	1.37 (0.98–1.92)

RC, reference category.

Boldface indicates statistical significance (*p < 0.05; **p < 0.01; ***p < 0.001).

a Adjusted for sex and age (only for age by sex and for sex by age).

### Suicide methods

The prevalence of BAC+ was highest in those who used hanging or suffocation as a suicide method (38.2%), followed by poisoning by other means (36.6%). Compared to hanging, odds of BAC+ were significantly lower for those who died by poisoning by drugs (AdjOR = 0.63, 95% CI 0.53–0.74), firearms and explosive (AdjOR = 0.78, 95% CI 0.63–0.97) or by other means (AdjOR = 0.61, 95% CI 0.52–0.72) (Table 1).

### Psychiatric and physical conditions and life events

Odds of BAC+ at the time of suicide were 26% lower in those who had a diagnosed psychiatric disorder (AdjOR = 0.74, 95% CI 0.66–0.82) (Table 1). More specifically, the BAC+ odds were reduced for those with psychotic (AdjOR = 0.33, 95% CI 0.25–0.43), bipolar (AdjOR = 0.75, 95% CI 0.58–0.96), anxiety disorders (AdjOR = 0.78, 95% CI 0.64–0.96) and unipolar depression (AdjOR = 0.89, 95% CI 0.80–1.00), and increased for those who were diagnosed with substance use disorders (AdjOR = 1.75, 95% CI 1.45–2.12). Those with a physical condition had also lower odds of BAC+ (AdjOR = 0.70, 95% CI 0.63–0.78). However, those with untreated mental health problems (AdjOR = 1.25, 95% CI 1.12–1.39) and of lifetime suicidal ideation (OR = 1.20, 95% CI 1.08–1.33) had higher odds of BAC+.

Odds of BAC+ were higher in those who had recent relationship conflict (AdjOR = 1.90, 95% CI 1.66–2.18), interpersonal conflict (AdjOR = 1.77, 95% CI 1.42–2.20), child custody dispute (AdjOR = 1.47, 95% CI 1.16–1.86), relationship separation (AdjOR = 1.29, 95% CI 1.15–1.45) and recent/pending unemployment (AdjOR = 1.23, 95% CI 1.03–1.47).

### Other drugs and substances in blood

It was more likely for BAC+ cases to have no medicine in blood at the time of suicide compared to BAC− group after adjusting for sex, age, unipolar depression, bipolar depression, anxiety, psychotic, substance abuse disorders and other psychiatric disorders (including all other psychiatric conditions reported) (AdjOR = 1.55; 95% CI 1.38–1.75) (Table 2). Nevertheless, it is important to note that 51.7% of suicides with BAC+ had at least one nervous system medicine (ATC N-class) in their blood similarly to BAC− group, with antidepressants (N6A – 27.6%) and anxiolytics (N5B – 24.1%) being the most common medicines. Antidepressants were the only drug group where there was no difference between BAC+ and BAC− groups, after adjusting for age and sex. Illegal drugs were somewhat more prevalent in the BAC+ group, but this difference did not persist after adjusting for age and sex. Not having any substances (other than alcohol in BAC+) was more likely in BAC+ group (AdjOR = 1.40; 95% CI 1.25–1.58) (Table 2).

**Table 2.** BAC+ and BAC− by different substances

	BAC+		BAC−			
	*N*	%	*N*	%	Crude OR (95% CI)	Adjusted OR (95% CI)^a^
Opioids (N2A)	279	12.8	986	21.6	**0.53** (0.46–6.62)***	**0.56** (0.48–0.65)***
Other analgesics (N2B, N2C)	234	10.7	683	15.0	0.68 (0.58–0.80)***	0.79 (0.67–0.93)**
Antipsychotics (N5A)	146	6.7	613	13.4	0.46 (0.38–0.56)***	0.58 (0.47–0.72)***
Anxiolytics (N5B)	526	24.1	1340	29.4	0.77 (0.68–0.86)***	0.85 (0.75–0.97)*
Hypnotics/sedatives (N5C)	280	12.8	1039	22.8	0.50 (0.43–0.58)***	0.57 (0.49–0.66)***
Antidepressants (N6A)	601	27.6	1517	33.2	0.77 (0.68–0.86)***	0.90 (0.79–1.03)
Other nervous system medicines (N1, N3, N4, N6B, N6D, N7)	173	7.9	619	13.6	0.55 (0.46–0.66)***	0.55 (0.46–0.66)***
Any nervous system medicine (all N group)	1127	51.7	2993	65.6	0.56 (0.51–0.62)***	0.66 (0.59–0.74)***
Other medicines (all other main groups)	288	13.2	901	19.7	0.62 (0.54–0.72)***	0.73 (0.63–0.85)***
No medicine	1001	45.9	1443	31.6	1.84 (1.65–2.04)***	1.55 (1.38–1.75)***
Illegal drugs	450	20.6	775	17.0	1.27 (1.12–1.45)***	1.08 (0.94–1.23)
Other substances^b^	32	1.5	130	2.8	0.51 (0.34–0.75)**	0.53 (0.36–0.79)**
No substances (except alcohol)	783	35.9	1171	25.7	1.62 (1.46–1.81)***	1.40 (1.25–1.58)***

Boldface indicates statistical significance (*p < 0.05; **p < 0.01; ***p < 0.001).

Drug codes refer to the ATC classification system.

a Adjusted for sex, age, unipolar depression, bipolar depression, anxiety, psychotic, substance abuse disorders and other psychiatric disorders (including all other psychiatric conditions reported).

b Mainly herbicides and pesticides.

### Logistic regression model

Forward Stepwise logistic regression analysis was performed to identify independent factors distinguishing between BAC+ and BAC−. Age group (highest in: 25–44 years), ethnicity (Indigenous), marital status (separated, divorced), suicide method (hanging), diagnosis of substance use, not having psychotic and other psychiatric disorder, lifetime suicidal ideation, relationship and interpersonal conflict and not having any nervous system drug or any other substances in blood at the time of suicide remained significant in the final model (*χ*^2^_(22)_ = 668.96, *p* < 0.001) and explained 13.2% of the variance in BAC+ odds (Table 3).

**Table 3.** Forward logistic regression model of BAC

		95% CI		
	OR	*L*	*U*	*p*-value
Age groups				
Below 25 years	0.62	0.52	0.74	**<0**.**001**
25–44 years	1 (RC)			
45–64 years	0.86	0.76	0.98	**0.025**
65+ years	0.33	0.27	0.42	**<0.001**
Ethnicity				
Indigenous	2.19	1.76	2.73	**<0.001**
Unknown	1.12	0.96	1.31	0.146
Other Australian	1 (RC)			
Marital status				
Never married/single	1.10	0.94	1.29	0.242
Separated	1.59	1.33	1.88	**<0.001**
Divorced	1.44	1.15	1.82	**0.002**
Widowed	0.88	0.58	1.34	0.559
Unknown	1.27	1.07	1.52	**0.007**
Married/*de facto*	1 (RC)			
Suicide methods				
Poisoning by drugs/medicine	0.73	0.62	0.87	**<0.001**
Poisoning by other means	1.13	0.94	1.36	0.194
Firearms/explosives	0.86	0.69	1.08	0.197
Other methods	0.73	0.62	0.87	**0.001**
Hanging/suffocation	1 (RC)			
Substance use disorders	2.05	1.67	2.52	**<0.001**
Psychotic disorders	0.34	0.26	0.46	**<0.001**
Other psychiatric disorder	0.74	0.59	0.92	**0.008**
Lifetime suicidal ideation	1.14	1.02	1.28	**0.024**
Relationship conflict	1.79	1.53	2.09	**<0.001**
Interpersonal conflict	1.58	1.25	1.99	**<0.001**
Any nervous system medicine in blood	0.68	0.60	0.76	**<0.001**
Other substances in blood^a^	0.51	0.34	0.76	**0.001**

Boldface indicates statistical significance (*p* < 0.05).

BAC− used as the reference group.

*χ*^2^(22) = 668.96, *p* *<* *0.001*; Nagelkerke *R*^2^ = 0.132.

a Mainly herbicides and pesticides.

## Discussion

While numerous investigations have analysed BAC in suicide cases, and their demographic distribution, only a small number of studies have examined psychosocial factors, such as psychiatric conditions and life events, to distinguish individuals whose suicide was impacted by alcohol use. To our knowledge, our study is the first comprehensive analysis of the co-ingestion of alcohol and different types of medicines and substances at the time of suicide. Our findings showed that approximately one-third (32.3%) of people who died by suicide in Queensland between 2004 and 2015 had a ‘positive’ BAC, which was considered as BAC ⩾ 0.05 g/dl. This result is comparable to a meta-analysis of 92 studies presenting an overall weighted mean of BAC positive in 33.6% of cases (Anestis *et al*., 2014; Conner, 2015). While we did not observe notable changes in BAC-positive suicide trends in Queensland in 2004–2015, some reduction in alcohol consumption was estimated during this time period in Australia (Australian Institute for Health and Welfare).

Our findings by sociodemographic variables such as age, sex and ethnicity reflected relatively similar patterns to earlier studies with the age group 25–44 having the highest odds of positive BAC (Kaplan *et al*., 2013; Lee *et al*., 2017). While our analyses also showed a higher prevalence of positive BAC in males compared to females (33.6 and 28.3%, respectively), sex did not remain in the final model to distinguish those who died by suicide under influences of alcohol.

Based on our findings, over half of Australian Indigenous who died by suicide were BAC+, and in the final model they had 2.2-times higher odds of BAC+ compared to other Australians who died by suicide. The problematic use of alcohol and a diagnosis of substance use disorder have been shown to be more prevalent among Aboriginal and Torres Strait Islander peoples who died by suicide compared to other Australian suicides in earlier studies (De Leo *et al*., 2012). Furthermore, it echoes also the US findings presenting that American Indian and Alaska Native had higher odds of positive BAC at the time of suicide compared to their Caucasian counterparts (Caetano *et al*., 2013; Kaplan *et al*., 2013).

Marital status remained significant in the final model reflecting that those who were separated and divorced were more likely under the influence of alcohol at the time of suicide. This also highlights the importance of recent life events, such as relationship conflicts and relationship separation, which could also be related to the use of alcohol to reduce the stress related to the separation. In combination with alcohol myopia, aggressive–impulsive behaviour and loss of barriers, this might culminate in suicidal behaviour. Furthermore, our earlier studies have shown that shame and alcohol and substance abuse in the previous year increase suicidal ideation in separated males and females (Kõlves *et al*., 2010) with separated males having the highest risk of suicide compared to other marital categories (Wyder *et al*., 2009).

While a few recent studies have shown no difference for mental health problems by BAC (Kaplan *et al*., 2013; Lee *et al*., 2017), our study showed that those with a psychiatric diagnosis were less likely to have a positive BAC compared to those without a diagnosed psychiatric disorder. More specifically, those with psychiatric conditions had lower odds of positive BAC, except substance use disorders, which showed higher odds. Nevertheless, those with undiagnosed mental disorders were more likely to have positive BAC. It could be suggested that alcohol might be used for self-medicating when facing life difficulties such as separation and that people with untreated mental health problems could be more likely to act on their suicidal ideation under the influence of alcohol, losing the barriers against it.

In the USA, there have been some indications that people who die by suicide under alcohol intoxication would be more likely to use highly lethal methods, such as firearms and hanging (Kaplan *et al*., 2013; Conner *et al*., 2014). In Australia, firearms are less available. The availability of suicide methods seems to be critical, in the absence of pre-planning, if a person becomes suicidal at the time of alcohol consumption. Our results appear to support that the prevalence of positive BAC was highest in cases with easily available and lethal means such as hanging or suffocation (38.2%) with 60% having positive BAC. Nevertheless, a large-scale study from Sweden showed that the poisoning had the highest positive BAC prevalence (Holmgren and Jones, 2010), and Finnish studies analysing fatal poisonings suggest that alcohol may increase lethality when co-ingested with medicines and lower levels of alcohol could be deadly (Koski *et al*., 2005). Nevertheless, we found that those who used poisoning by drugs and firearms were less likely to have positive BAC compared to hanging/suffocation cases, with less than a quarter (23.7%) of all drug overdoses involving also alcohol.

Our analysis into different substances showed that nervous system medicines were less likely to be ingested with alcohol compared to those who did not consume alcohol. Only a quarter of the group that did not have alcohol involvement did not have any substances in the blood (17.4% out of all suicides). Interestingly, analysis of non-fatal intentional self-poisonings from Australia found that psychotropic drugs were less likely to be consumed with alcohol (Chitty *et al*., 2017). Our analysis showed that the risk of ingestion of alcohol with all drug groups, except antidepressants and illegal drugs, did remain significantly lower after adjusting for sex, age and different psychiatric diagnoses compared to those who did not consume alcohol before suicide. Considering that our investigation focused on medicines present in the blood rather than being prescribed or taken regularly, it could also be suggested that taking highly sedating drugs may be sufficient to remove barriers to intentional self-harm even in the absence of alcohol. Nevertheless, lethality in the drug overdoses needs further examination.

Some limitations have to be noted. Our analysis is based on a suicide register and therefore does not have a control/comparison group. Nevertheless, a few studies including other types of death have not shown a significant difference in the use of alcohol before death. For example, an Australian study showed that there was no significant difference in the prevalence of positive BAC homicides and violent suicides (not inclusive of drug overdoses) (Darke *et al*., 2009). Our study included 92% of all suicide cases in Queensland as 3.9% were missing a toxicology report and due to several reasons (e.g., body decomposition level) and in 4.1% of suicide it was not feasible to measure BAC level. While the QSR is a comprehensive suicide surveillance system, it is reliant on external data sources such as police/coronial information along with the conjectures of relatives. Consequently, the accuracy of information depends on the quality of the investigating process. In addition, the precision of data may also be impacted by the informant's lack of knowledge of different aspects of the deceased's life (e.g., presence of physical or psychiatric diagnosis).

### Implications

The results suggest that people who die by suicide while under the influence of alcohol are more likely to have relationship problems, such as stress caused by separation or divorce, and they are less likely to have previous mental health diagnosis or take any nervous system drugs. Under these conditions, alcohol consumption might increase the probability of a number of potential mechanisms, such as an increase in aggressive behaviour, loss of barriers, but also alcohol myopia. This highlights the importance of counselling for people going through a separation, whilst also helping them to control alcohol intake, which could be contributing to suicidal behaviour. Nevertheless, similarly to earlier studies, people with substance use problems have a higher risk of also using alcohol at the time of their suicides, which reflects the need for timely diagnosis, improved and available treatment, and screening for suicidality (Kõlves *et al*., 2017). Witt and Lubman (2018) highlighted the need for alcohol and other drug services in Australia to have resources for skilled staff to identify people at risk of suicide, provide proper interventions, and effective partnerships with other services to provide support and referral pathways. Another important aspect is their accessibility. While the Aboriginal and Torres Strait Islanders are over seven-times more likely to have received treatment for alcohol than non-Indigenous Australians (AIHW), access to the services is an issue in rural and remote areas with the travel time over 1 h being the main reason for closing the treatment (AIHW).

While the links between suicide and alcohol consumption on the aggregate level would need more robust analyses and were out of the scope of the current study, some changes in alcohol policies should be also considered. Studies from the former republics of the Soviet Union have shown that strict alcohol policy at the time of *Perestroika* (restructuring) in the mid-1980s were related specifically to the drop in suicides with positive BACs and no change in sober suicides (Nemtsov, 2003; Värnik *et al*., 2007). In addition to rather extreme measures employed (e.g. destroying vineyards, closing factories), the measures included limiting access to alcohol by increasing alcohol prices, campaign to change attitudes and improving treatment practices (Värnik *et al*., 2007) are among the strategies encouraged by the World Health Organization (2018) to reduce harmful alcohol use.

## Data

The Queensland Suicide Register, the database used in the current study, has no public access due to the sensitivity of the suicide data.
